# Source-specific nitrate and nitrite intake and associations with gastric cancer in the Danish Diet, Cancer and Health Cohort

**DOI:** 10.1007/s10654-026-01390-6

**Published:** 2026-04-30

**Authors:** Dorit W. Erichsen, Cecilie Kyrø, Pratik Pokharel, Susanne Rosthøj, Catherine P. Bondonno, Liezhou Zhong, Jörg Schullehner, Torben Sigsgaard, Christina Dahl, Peter Fjeldstad Hendriksen, Frederik Dalgaard, Ole Raaschou-Nielsen, Jonathan M. Hodgson, Christina C. Dahm, Anja Olsen, Anne Tjønneland, Nicola P. Bondonno

**Affiliations:** 1 Danish Cancer Institute, Copenhagen, Denmark; 2 Nutrition and Health Innovation Research Institute, School of Medical and Health Sciences, Edith Cowan University, Perth, Australia; 3 Medical School, The University of Western Australia, Royal Perth Hospital, Perth, WA Australia; 4 Department of Geochemistry, Geological Survey of Denmark and Greenland, Aarhus, Denmark; 5 Department of Public Health, Aarhus University, Aarhus, Denmark; 6 Department of Cardiology, Herlev & Gentofte University Hospital, Copenhagen, Denmark; 7 Department of Environmental Science, Aarhus University, Roskilde, Denmark; 8 Department of Public Health, Faculty of Health and Medical Sciences, University of Copenhagen, Copenhagen, Denmark; 9 Institute of Agriculture, The University of Western Australia, Royal Perth Hospital, Perth, WA Australia; 10 Danish Big Data Centre for Environment and Health (BERTHA), Aarhus University, Aarhus, Denmark

**Keywords:** Diet, Nitrate, Nitrite, Drinking water, Gastric cancer

## Abstract

Nitrate and nitrite, present in foods and drinking water, may contribute to gastric cancer (GC) through the formation of carcinogenic *N*-nitrosamines, but risks likely vary by source and endogenous nitrosating factors. In the Danish Diet, Cancer, and Health Cohort, source-specific nitrate and nitrite intakes (plant sources, naturally-occurring animal sources, additive-permitted meat sources, and drinking water) were estimated from a food frequency questionnaire using food databases and water monitoring data. Associations with GC, identified via the Danish Cancer Registry, were examined using multivariable-adjusted Cox proportional hazards models. Analyses were stratified by GC risk factors and factors hypothesized to influence endogenous *N*-nitrosamine formation to explore potential effect modification. Among 54,610 participants, 260 incident cases of GC (120 cardia, 59 non-cardia, and 81 overlapping/unclassified) were identified over 27 years. Plant sourced nitrite intake was associated with lower GC rates [HR_Q5vQ1_ (95%CI): 0.63 (0.41, 0.96)]. Conversely, intakes of nitrite from naturally-occurring animal sources [HR_Q4vQ1_ 1.55 (1.01, 2.38)]; and both nitrate and nitrite from additive-permitted meat sources were associated with higher rates of GC [HR_Q5vQ1_ 3.12 (1.09, 8.88) and 1.94 (1.19, 3.15), respectively], with higher point estimates for non-cardia GC. Nitrate from tap water was associated with higher rates of cardia GC rates [HR_Q5vsQ1_ 1.95 (1.11, 3.43)]. Nitrate and nitrite intakes demonstrated heterogeneous associations with GC incidence according to source and anatomical subsite. These findings warrant the re-evaluation of legislation on nitrate and nitrite additives in processed meats and threshold levels for nitrate in drinking water, as well as further investigation into nitrite from animal-sourced foods.

**Supplementary Information:**

The online version contains supplementary material available at 10.1007/s10654-026-01390-6.

## Introduction

Gastric cancer (GC) accounts for nearly one million new cases annually worldwide and ranks fifth in terms of both incidence and mortality [1, 2]. With a poor overall prognosis and a five-year survival of less than 30%, GC represents a major global health burden [2]. As a disease of older adults, its incidence is of particular concern in the context of rising life expectancy [3–5].

GC is a heterogeneous disease comprising distinct subsites. Cardia and non-cardia GC differ in both pathogenesis and etiology, but modifiable risk factors and underlying mechanisms driving these differences are not yet fully understood. Chronic infections, smoking, adiposity, and several dietary factors are implicated in the etiology of GC [6]. Among the latter, nitrate and nitrite are of particular interest due to their ability to form carcinogenic *N*-nitrosamines both endogenously and exogenously [7], a mechanism central to established etiologic models of gastric carcinogenesis, first hypothesized in 1975 [8]. Nitrate and nitrite are found ubiquitously in the diet as they occur in drinking water, plant-sourced foods, and animal-sourced foods, particularly processed meats where they are added as preservatives [9]. Following ingestion, approximately 25% of circulating nitrate is concentrated in saliva where oral bacteria reduce it to nitrite [10]. In the acidic stomach, nitrite can react with secondary amines to form *N*-nitrosamines (Supplementary Fig. 1). These potent carcinogens, first identified in 1956 [11], induce tumors in animal models, and are classified by the International Agency for Research on Cancer (IARC) as probably (Group 2 A) or possibly (Group 2B) carcinogenic to humans under conditions that promote endogenous nitrosation [12].

Epidemiological research on nitrate and nitrite intake as cancer risk factors has expanded in recent years, particularly for colorectal cancer [13]. Findings for GC remain limited and inconsistent [14, 15]. Most studies have treated GC as a single entity, limiting insights into potential etiologic heterogeneity by tumor location. In addition, few studies have differentiated nitrate and nitrite intake by source, despite intake source being a critical determinant of their biological fate, as recognized in long-established frameworks of gastric carcinogenesis [8]. Nitrate from vegetables is often accompanied by bioactive components that may inhibit the formation of carcinogenic *N*-nitrosamines, allowing nitrate to be reduced to the beneficial compound, nitric oxide. Conversely, nitrate and nitrite from animal sources (especially meat sources undergoing nitrate/nitrite preservation) and contaminated drinking water may be more likely to contribute to endogenous nitrosation when promoting factors such as smoking are present [16, 17]. Supporting biological plausibility, animal and mechanistic studies show that nitrate-derived *N*-nitrosamines can induce DNA damage and cellular transformation, key processes in gastric carcinogenesis [18].

This study aimed to examine associations between source-specific nitrate and nitrite intake (plant sources, naturally-occurring animal sources, additive-permitted meat sources, and drinking water) and GC (total and subsites) in a large prospective cohort. A secondary aim was to explore potential effect modification of associations by stratifying by established GC risk factors and factors influencing endogenous *N*-nitrosamine formation.

## Methods

### Study population

This study makes use of data from the Danish Diet, Cancer and Health (DCH) Cohort, comprising 57,053 cancer-free adults aged 50–65 years from Copenhagen and Aarhus metropolitan areas recruited between 1993 and 1997 [19]. Using Denmark’s unique personal identification system, cohort participants were linked to a series of national registries [20–26] to obtain comprehensive covariate, follow-up and outcome data.

### Source-specific nitrate and nitrite intakes

Dietary nitrate and nitrite intake was estimated using a validated semi-quantitative food frequency questionnaire (FFQ) completed by participants, which captured average consumption of 192 foods and beverages over the preceding 12 months prior to enrollment [27–29]. All food and beverages in the FFQ were categorized into four exposure groups for the purpose of source-specific analyses: plant-sourced foods (fruits, vegetables, legumes, whole grains); animal-sourced foods where nitrate and nitrite are naturally occurring (red meat, poultry, offal, dairy, eggs, fish, and seafood); additive-permitted meat sources (processed meats including bacon, ham, salami, and sausage etc.), and drinking water [30]. Detailed methodology, including correlation analyses, have been described in detail previously [13, 30].

#### Calculation of plant- and animal-sourced nitrate and nitrite intakes

Estimates of the nitrate and nitrite contents of all foods and beverages in the FFQ were obtained from published databases and government monitoring programmes [9, 31], totaling 130,018 complete entries for nitrate and 22,104 complete entries for nitrite [9]. Preference was given to values from Danish sources. Intakes were calculated by multiplying consumption quantities (g/day) by median nitrate/nitrite values (mg/g), adjusting for 50% nitrate reduction in boiled vegetables [9].

#### Calculation of water-sourced nitrate intakes

Nitrate from drinking water was calculated using the publicly accessible national geodatabase Jupiter, which holds information on public waterworks, private wells and drinking water quality over decades [32, 33]. Comprehensive details on water nitrate calculations for this cohort have been described previously [30]. Briefly, we estimated the nitrate content of drinking water for each participant by tracing residential histories in the Civil Registration System and spatiotemporally linking the information on residency time period and location with the estimated nitrate content within each addresses’ water supply area from the same time period [30, 33]. To estimate baseline intakes of tap water-sourced nitrate, intakes of tap water were obtained from the FFQ and multiplied by the time-weighted average of the nitrate concentration at every address each cohort participant lived at in the 12 months prior to their enrolment into the study. Participants using private wells were excluded due to lower measurement frequency [33]. As constituents in tea, coffee, and water added to fruit syrup might influence the formation of *N*-nitrosamines, the primary analysis included intake of tap water only, and bottled water consumption was not considered given negligible intake in Denmark.

### Gastric cancer outcomes

The primary outcome was incident GC (ICD-10: C16) as first primary cancer, identified via the Danish Cancer Registry [20], and examined by the main anatomical subsites: tumors in the cardia were grouped as cardia GC (ICD-10: C16.0), and tumors in the distal part of the stomach were grouped as non-cardia GC (ICD-10: C16.1-C16.6). Overlapping tumors (C16.8) or a GC diagnosis with unspecified tumor site (C16.9) were grouped as overlapping/unspecified GC.

### Covariates

Information on age, sex, smoking status (never, former, current), smoking history (pack years) and physical activity (metabolic equivalent of task (MET) score) was obtained from self-administered questionnaires completed by participants at time of enrolment. Intake of alcohol (g/d), total flavonoids (mg/d; calculated using the Phenol-Explorer database [34]), folate (µg/d), vitamin C (µg/d), and vitamin E (mg α tocopherol equivalents/d) were estimated from the FFQ. Dietary flavonoids, folate, vitamin C, and vitamin E intakes were grouped into tertiles. BMI (kg/m^2^) was computed from anthropometric measurements taken by trained personnel at study centers at time of enrolment. Education level was obtained from the Education Registry, and categorized as short (≤7 years), medium (8-10–12 years), or high (11 years). Information on domestic status was obtained from the Civil Registration System [23]. To capture Helicobacter pylori (*H. pylori*) infection, a major risk factor for especially non-cardia GC, we used a proxy approach commonly applied in epidemiological studies lacking serological testing for antibodies or other direct diagnostic data [35]. Specifically, we defined a history of gastric ulcers as a proxy for *H. pylori* infection, based on well-documented associations where *H. pylori* infection account for up to 90% of gastric ulcers globally [36, 37]. The variable was constructed using diagnoses recorded in the Danish National Patient Registry for peptic ulcers (ICD-8: 531–534; ICD-10: K25-K28) and gastritis (ICD-8: 535; ICD-10: K29) [22, 25], or records of *H. pylori* eradication treatment in the Danish National Prescription Registry [21], defined as the prescription, within 30 days, of either (i) a proton pump inhibitor (PPI; ATC: A02BC) with amoxicillin (J01CA04) and clarithromycin (J01FA09), or (ii) a PPI with metronidazole (P01AB01) and clarithromycin.

### Statistical analysis

Baseline characteristics were summarized for the whole cohort and according to quintiles of nitrate intake from plant-sources, animal sources where nitrate/nitrite are naturally occurring, meat sources where nitrate/nitrite are permitted additives, and tap water, respectively. The median follow-up time was determined using the reverse Kaplan-Meier estimator. Associations between each exposure and GC and subsites were analyzed using Cox proportional hazards models with age as the underlying timescale and delayed entry from time of enrollment. Participants were followed until the earliest of any cancer diagnosis, death, emigration, or end of follow-up (31 December 2020). Exposure variables were grouped into quintiles (Q1-Q5), with Q1 as reference group, and additionally modelled using a logarithmic transformation base 2 to address right-skewed distributions. For exposures with zero values, half of the smallest non-zero value for that respective exposure variable was added before transformation, and all quantitative covariates were cubic root transformed to mitigate skewness.

To provide transparency in confounding control, three models with progressive adjustment were used: a minimally adjusted model (Model 1), a sociodemographically-adjusted model (Model 2), and a dietary-adjusted model (Model 3). Model 1 included sex. Model 2 included sex, BMI, smoking status, smoking packyears, alcohol consumption, education level, physical activity level and domestic status. To account for observed differences in underlying dietary patterns in the cohort [30], Model 3 applied the ‘all-components’ approach used in nutritional epidemiology [38, 39], which adjusts for all energy-contributing food groups excluding the exposure food group. Specifically, Model 3 included all covariates from Model 2 plus different sets of dietary variables depending on the exposure: (a) when plant-sourced nitrate/nitrite were the exposures of interest, intakes of red meat, processed meat, poultry, dairy, fish, refined grains, sugar and confectionery, soft drinks, coffee, and tea were included to control for animal-sourced and other dietary nitrate/nitrite contributions; (b) when animal-sourced nitrate/nitrite were the exposures of interest, intakes of wholegrains, refined grains, vegetables, fruits, vegetable oils, sugar and confectionery, soft drinks, coffee, and tea were included to control for plant-sourced and other dietary nitrate/nitrite contributions; and (c) when tap water nitrate (mg/d) was the exposure of interest, intakes of wholegrains, refined grains, red meat, processed meat, poultry, dairy, fish, vegetables, fruits, vegetable oils, sugar and confectionery, coffee, tea, and soft drinks were included to control for all dietary nitrate/nitrite sources. Covariates were chosen a priori based on knowledge of relationships with the exposures (nitrate/nitrite intake) and outcome (rate of GC) [30]. Given the limited number of cases and the extensive parameterization required for Model 3, Model 2 serves as our primary analysis model. However, Model 3 results are also presented in the tables as they provide important control for dietary confounding patterns.

To examine potential effect modification, using Model 2, analyses were stratified by known risk factors for GC: sex; smoking status (current and former smokers vs. never smokers), gastric ulcer as a proxy for *H. pylori* infection (yes/no); abdominal obesity (waist-to-hip ratio ≥ 0.90 for men and ≥ 0.85 for women; yes/no) [40], meeting the recommended maximum intake of alcohol (≤ 14 units/week for men and ≤ 7 units/week for women; yes/no), as well as factors known to influence endogenous formation of *N*-nitrosamines (Supplementary Fig. 1): high versus low intakes of vitamin C, vitamin E, folate, flavonoids, and red meat (tertile 3 vs. tertile 1 for all). Dietary exposures were modeled on the log₂ scale to reflect hypothesized logarithmic associations, while drinking water nitrate was modelled as quintile 5 versus quintile 1 because we hypothesized higher risk only at the highest exposure levels.

In a supplementary analysis, drinking water nitrate was treated as a time-varying exposure. At each age, exposure was estimated as the cumulative average nitrate concentration in the drinking water supplied to the participant’s residential addresses over 15 years prior to a diagnosis of GC or administrative censoring.

Analyses were undertaken using R version 4.3.1 [41] and SAS version 9.4 [42].

## Results

Among 57,053 participants enrolled in the DCH Cohort, we excluded 590 ineligible participants and 1,853 participants who had missing data or were supplied by private wells, yielding a final analytical cohort of 54,610 participants (Supplementary Fig. 2). The median age at baseline was 56 years (IQR: 52–60) and 47.5% of the cohort participants were men. Over a median follow-up of 24.7 years (IQR: 24.1–25.3; maximum 27 years), 260 incident cases of GC were identified, comprised of 120 cardia GC, 59 non-cardia GC, and 81 overlapping or unclassified GC tumors. More men (*n* = 155) than women (*n* = 105) were diagnosed with GC in the cohort.

### Baseline characteristics

Individuals with the highest intake of plant-sourced nitrate were more likely to be female, physically active, have never smoked, to be cohabitating and to have attained higher education levels (Table 1). A broadly similar pattern was observed among those with the highest intakes of nitrate from naturally-occurring animal sources, although participants in the lower quintile in this category were more often female. In contrast, participants with high intakes of nitrate from additive-permitted meat sources were predominantly male, more frequently current smokers, consumed more alcohol, and had lower educational attainment. For intakes of nitrate from tap water, participants in the highest quintile were more likely to be female, have higher physical activity levels, never-smoking status, lower alcohol consumption, lower education level, and more likely to live alone.

**Table 1:** Baseline characteristics of the study population by quintiles of source-specific nitrate intakes

	Total participants	Plant sourced nitrate		Naturally-occurring animal sourced nitrate		Additive-permitted meat sourced nitrate		Tap water sourced nitrate	
	*N* = 54,610	Q1 (*n* = 10,922)	Q5 (*n* = 10,922)	Q1 (*n* = 10,922)	Q5 (*n* = 10,922)	Q1 (*n* = 10,922)	Q5 (*n* = 10,922)	Q1 (*n* = 10,922)	Q5 (*n* = 10,918)
*Nitrate intake (mg/d)*:									
Plant sources	43.89 [31.04, 59.61]	22.19 [18.08, 25.38]	76.69 [69.75, 88.23]	35.39 [24.18, 51.18]	52.94 [39.30, 69.82]	47.75 [32.84, 65.97]	42.28 [30.21, 57.39]	38.12 [26.66, 51.91]	47.24 [33.62, 64.41]
Naturall-occurring animal sources	5.42 [3.72, 8.07]	4.15 [3.00, 6.30]	6.75 [4.43, 9.56]	2.74 [2.29, 3.09]	10.69 [9.48, 13.45]	4.80 [3.03, 7.60]	5.93 [4.32, 8.62]	5.12 [3.70, 7.58]	5.47 [3.57, 8.18]
Additive-permitted meat sources	0.28 [0.16, 0.48]	0.30 [0.17, 0.50]	0.25 [0.13, 0.44]	0.21 [0.12, 0.36]	0.33 [0.19, 0.54]	0.09 [0.06, 0.12]	0.73 [0.62, 0.95]	0.35 [0.20, 0.57]	0.24 [0.13, 0.41]
Tap water	0.79 [0.25, 1.74]	0.56 [0.13, 1.45]	1.01 [0.42, 1.94]	0.88 [0.26, 1.88]	0.86 [0.32, 1.81]	1.03 [0.41, 1.96]	0.51 [0.15, 1.30]	0.04 [0.00, 0.10]	3.26 [2.57, 4.84]
Nitrate concentration in tap water supplying the household (mg/L)	1.99 [1.20, 2.85]	2.03 [1.22, 2.91]	1.99 [1.21, 2.70]	2.03 [1.23, 2.86]	1.98 [1.19, 2.78]	2.02 [1.29, 2.85]	1.95 [1.17, 2.85]	1.68 [0.87, 2.16]	3.02 [2.17, 5.40]
Age (years)	56.0 [52.0, 60.0]	56.0 [53.0, 60.0]	55.0 [52.0, 60.0]	56.0 [52.0, 60.0]	56.0 [52.0, 60.0]	56.0 [53.0, 60.0]	55.0 [52.0, 60.0]	55.0 [52.0, 59.0]	56.0 [52.0, 60.0]
Sex (male)	25,965 (47.5)	5507 (50.4)	4548 (41.6)	3514 (32.2)	5614 (51.4)	2228 (20.4)	8774 (80.3)	7619 (69.8)	3235 (29.6)
BMI (kg/m2)	25.5 [23.3, 28.2]	26.0 [23.6, 28.9]	25.0 [22.9, 27.5]	25.2 [22.9, 27.9]	25.6 [23.3, 28.2]	24.8 [22.6, 27.5]	26.1 [23.8, 28.8]	25.7 [23.5, 28.2]	25.6 [23.2, 28.6]
MET score	56.5 [37.0, 85.0]	48.8 [30.5, 75.8]	64.5 [43.0, 93.5]	52.5 [33.8, 79.5]	61.5 [40.0, 91.0]	57.5 [38.0, 86.0]	57.0 [35.5, 88.5]	51.8 [33.0, 79.5]	61.0 [39.5, 91.0]
Energy (kcal/d)	2270.6 [1877.9, 2718.6]	1946.2 [1598.2, 2338.0]	2573.6 [2173.1, 3059.5]	1816.9 [1537.1, 2148.6]	2661.6 [2258.3, 3144.3]	1916.3 [1588.6, 2283.7]	2791.8 [2375.8, 3258.9]	2349.2 [1952.3, 2794.8]	2183.0 [1804.3, 2627.5]
Domestic status (single)	14,185 (26.0)	3519 (32.2)	2975 (27.2)	3310 (30.3)	3074 (28.1)	4017 (36.8)	2613 (23.9)	2534 (23.2)	3518 (32.2)
*Education level*									
≤ 7 year	9990 (18.3)	2775 (25.4)	1436 (13.1)	2298 (21.0)	1947 (17.8)	1691 (15.5)	2478 (22.7)	2011 (18.4)	2329 (21.3)
8–10 years	29,828 (54.6)	6565 (60.1)	5124 (46.9)	6276 (57.5)	5488 (50.2)	5681 (52.0)	6118 (56.0)	5990 (54.8)	6182 (56.6)
≥ 11 years	14,792 (27.1)	1582 (14.5)	4362 (39.9)	2348 (21.5)	3487 (31.9)	3550 (32.5)	2326 (21.3)	2921 (26.7)	2407 (22.0)
*Smoking*									
Never	19,255 (35.3)	3046 (27.9)	4331 (39.7)	3793 (34.7)	4088 (37.4)	4634 (42.4)	2790 (25.5)	3175 (29.1)	4113 (37.7)
Former	15,619 (28.6)	2578 (23.6)	3582 (32.8)	2876 (26.3)	3267 (29.9)	3072 (28.1)	3161 (28.9)	3049 (27.9)	3014 (27.6)
Current	19,736 (36.1)	5298 (48.5)	3009 (27.5)	4253 (38.9)	3567 (32.7)	3216 (29.4)	4971 (45.5)	4698 (43.0)	3791 (34.7)
Smoking intensity (pack-years)	9.4 [0.0, 26.4]	18.6 [0.0, 32.0]	4.0 [0.0, 20.0]	10.0 [0.0, 26.2]	7.2 [0.0, 24.5]	2.9 [0.0, 19.0]	20.0 [0.0, 35.0]	17.1 [0.0, 33.0]	6.7 [0.0, 23.8]
*Alcohol intake*									
Alcohol (g/d)	12.9 [5.9, 31.1]	12.1 [3.6, 32.3]	12.7 [6.0, 29.3]	11.0 [3.3, 26.9]	13.3 [6.3, 30.5]	10.3 [3.2, 21.0]	16.5 [7.4, 36.8]	16.0 [7.0, 36.6]	11.2 [3.9, 23.0]
Below recommended limits	32,463 (59.4)	6636 (60.8)	6471 (59.2)	6701 (61.4)	6536 (59.9)	6780 (62.1)	6414 (58.7)	6196 (56.7)	6735 (61.7)

Table 1 presents baseline characteristics of the study population by first and fifth quintiles of source-specific nitrate intakes

Data is expressed as median [IQR] or n (%)

BMI, body mass index; MET, metabolic equivalent (determined from physical activity questionnaire); alcohol intake below the recommended maximum intake (male: ≤14 units/week and female: ≤7 units/week)

### Associations between source-specific nitrate and nitrite intakes and incident gastric cancer

For both plant-sourced nitrate and nitrite, we observed an overall pattern of higher intakes being associated with lower rates of cardia and non-cardia GC (Table 2). However, this was only statistically significant for plant-sourced nitrite intakes and overall GC [HR_Q5vQ1_ 0.63 (0.41, 0.96); Model 2].

**Table 2:** Hazards ratio of gastric cancer and subtypes by quintiles of nitrate and nitrite intake from plant sources

	Q1 *n* = 10,922	Q2 *n* = 10,922	Q3 *n* = 10,922	Q4 *n* = 10,922	Q5 *n* = 10,922	Log transformed *N* = 54,610
**Nitrate intake from plant sources**						
*Intake (mg/d)*	22.19 [18.08, 25.38]	33.70 [31.04, 36.29]	43.89 [41.32, 46.58]	55.83 [52.42, 59.61]	76.69 [69.75, 88.23]	43.89 [31.04, 59.61]
*Gastric cancer (combined)*						
No. of events	65	52	59	43	41	260
Model 1	Ref	0.77 (0.54, 1.11)	0.87 (0.61, 1.24)	0.63 (0.43, 0.93)	0.62 (0.42, 0.91)	0.83 (0.70, 0.98)
Model 2	Ref	0.80 (0.56, 1.16)	0.96 (0.67, 1.38)	0.72 (0.49, 1.07)	0.72 (0.48, 1.08)	0.90 (0.75, 1.07)
Model 3	Ref	0.80 (0.55, 1.16)	0.96 (0.66, 1.39)	0.72 (0.48, 1.09)	0.73 (0.48, 1.12)	0.91 (0.75, 1.10)
*Cardia gastric cancer*						
No. of events	31	26	27	17	19	120
Model 1	Ref	0.80 (0.48, 1.35)	0.85 (0.51, 1.42)	0.53 (0.29, 0.96)	0.62 (0.35, 1.10)	0.79 (0.62, 1.01)
Model 2	Ref	0.85 (0.50, 1.43)	0.96 (0.57, 1.62)	0.64 (0.35, 1.17)	0.79 (0.44, 1.42)	0.89 (0.68, 1.15)
Model 3	Ref	0.89 (0.52, 1.51)	1.03 (0.60, 1.77)	0.71 (0.38, 1.32)	0.89 (0.48, 1.66)	0.95 (0.72, 1.26)
*Non-cardia gastric cancer*						
No. of events	11	12	14	13	9	59
Model 1	Ref	1.05 (0.46, 2.39)	1.21 (0.55, 2.67)	1.12 (0.50, 2.49)	0.77 (0.32, 1.86)	0.96 (0.66, 1.38)
Model 2	Ref	1.05 (0.46, 2.38)	1.24 (0.56, 2.75)	1.15 (0.51, 2.63)	0.81 (0.33, 2.01)	0.98 (0.67, 1.45)
Model 3	Ref	0.93 (0.40, 2.13)	1.03 (0.45, 2.35)	0.92 (0.39, 2.15)	0.60 (0.23, 1.58)	0.84 (0.55, 1.29)
*Overlapping or unclassified gastric cancer*						
No. of events	23	14	18	13	13	81
Model 1	Ref	0.59 (0.30, 1.15)	0.75 (0.40, 1.39)	0.53 (0.27, 1.06)	0.53 (0.27, 1.05)	0.80 (0.59, 1.08)
Model 2	Ref	0.62 (0.32, 1.21)	0.82 (0.44, 1.54)	0.60 (0.30, 1.20)	0.60 (0.29, 1.21)	0.86 (0.63, 1.18)
Model 3	Ref	0.61 (0.31, 1.21)	0.82 (0.43, 1.56)	0.60 (0.29, 1.23)	0.61 (0.29, 1.29)	0.88 (0.62, 1.23)
**Nitrite intake from plant sources**						
*Intake (mg/d)*	0.39 [0.32, 0.45]	0.61 [0.56, 0.66]	0.80 [0.75, 0.85]	1.04 [0.97, 1.12]	1.46 [1.31, 1.74]	0.80 [0.56, 1.12]
*Gastric cancer (combined)*						
No. of events	66	57	52	49	36	260
Model 1	Ref	0.84 (0.59, 1.20)	0.76 (0.53, 1.09)	0.71 (0.49, 1.03)	0.53 (0.35, 0.79)	0.80 (0.68, 0.94)
Model 2	Ref	0.92 (0.64, 1.31)	0.86 (0.60, 1.25)	0.83 (0.57, 1.22)	0.63 (0.41, 0.96)	0.88 (0.74, 1.03)
Model 3	Ref	0.91 (0.63, 1.31)	0.85 (0.58, 1.24)	0.83 (0.56, 1.22)	0.62 (0.40, 0.97)	0.87 (0.73, 1.04)
*Cardia gastric cancer*						
No. of events	31	26	27	17	19	120
Model 1	Ref	0.91 (0.55, 1.53)	0.84 (0.50, 1.42)	0.62 (0.35, 1.10)	0.56 (0.31, 1.01)	0.77 (0.61, 0.97)
Model 2	Ref	1.02 (0.61, 1.71)	1.00 (0.58, 1.70)	0.78 (0.43, 1.39)	0.74 (0.40, 1.36)	0.87 (0.68, 1.11)
Model 3	Ref	1.07 (0.64, 1.82)	1.08 (0.63, 1.87)	0.86 (0.47, 1.57)	0.83 (0.44, 1.57)	0.92 (0.71, 1.19)
*Non-cardia gastric cancer*						
No. of events	11	12	14	13	9	59
Model 1	Ref	0.83 (0.38, 1.79)	0.82 (0.38, 1.77)	0.95 (0.45, 1.99)	0.47 (0.19, 1.17)	0.86 (0.62, 1.21)
Model 2	Ref	0.84 (0.39, 1.84)	0.84 (0.38, 1.84)	0.97 (0.45, 2.08)	0.49 (0.19, 1.24)	0.88 (0.62, 1.25)
Model 3	Ref	0.73 (0.33, 1.60)	0.67 (0.30, 1.49)	0.73 (0.33, 1.64)	0.34 (0.13, 0.92)	0.75 (0.50, 1.10)
*Overlapping or unclassified gastric cancer*						
No. of events	23	14	18	13	13	81
Model 1	Ref	0.75 (0.40, 1.40)	0.61 (0.31, 1.18)	0.69 (0.36, 1.31)	0.51 (0.25, 1.04)	0.81 (0.61, 1.08)
Model 2	Ref	0.83 (0.44, 1.57)	0.70 (0.35, 1.38)	0.79 (0.41, 1.54)	0.60 (0.29, 1.24)	0.88 (0.66, 1.18)
Model 3	Ref	0.81 (0.43, 1.56)	0.69 (0.34, 1.38)	0.78 (0.39, 1.55)	0.59 (0.27, 1.26)	0.88 (0.65, 1.21)

Table 2 presents the rate of gastric cancer and subsites over 27 years of follow-up by intake of nitrate and nitrite from plant sources

Hazard ratios (95% CIs) were estimated from Cox proportional hazards models with age as the underlying time scale. Nitrate and nitrite intakes were modelled categorically (quantiles) and as a logarithmic transformation base 2, where the hazard ratio represents the relative change in hazard associated with each doubling of the exposure (mg/d)

Model 1 included sex; Model 2 included sex, BMI, smoking status, smoking packyears, alcohol consumption, education level, physical activity level and living situation; Model 3 included all covariates in Model 2 and dietary confounders relevant for intake of nitrate and nitrite from plant sources as exposure (intake of red meat, processed meat, poultry, dairy, fish, sugar and confectionary, soft drinks, refined grains, coffee, and tea)

Exposure intakes are median [IQR]. Quintiles were defined based on the distribution of the exposure within the study population, with cutoff points at the 20th, 40th, 60th, and 80th percentiles

No associations were identified for nitrate naturally-occurring in animal sources and GC rates, either overall or across subsites (Table 3). For nitrite, however, we observed a higher rate of non-cardia GC in quintiles 4 and 5 [HR_Q4vQ1_ 2.98 (1.15, 7.71) and HR_Q5vQ1_ 2.42 (0.89, 6.57); Model 2], but no associations for cardia GC.

**Table 3:** Hazards ratio of gastric cancer and subtypes by quintiles of nitrate and nitrite intake from naturally-occurring animal sources

	Q1 *n* = 10,922	Q2 *n* = 10,922	Q3 *n* = 10,922	Q4 *n* = 10,922	Q5 *n* = 10,922	Log transformed *N* = 54,610
**Nitrate intake from naturally-occurring animal sources**						
*Intake (mg/d)*	2.74 [2.29–3.09]	4.02 [3.72–4.34]	5.42 [5.02–5.88]	7.50 [6.95–8.07]	10.69 [9.48–13.45]	5.42 [3.72, 8.07]
*Gastric cancer (combined)*						
No. of events	49	54	60	44	53	260
Model 1	Ref	1.00 (0.68, 1.48)	1.09 (0.74, 1.60)	0.83 (0.55, 1.25)	0.96 (0.65, 1.42)	0.94 (0.80, 1.10)
Model 2	Ref	1.01 (0.68, 1.49)	1.11 (0.75, 1.63)	0.88 (0.59, 1.33)	1.03 (0.69, 1.53)	0.97 (0.83, 1.13)
Model 3	Ref	0.99 (0.67, 1.47)	1.09 (0.74, 1.61)	0.88 (0.58, 1.33)	1.02 (0.67, 1.54)	0.96 (0.81, 1.13)
*Cardia gastric cancer*						
No. of events	17	24	30	26	23	120
Model 1	Ref	1.15 (0.62, 2.15)	1.37 (0.75, 2.49)	1.31 (0.71, 2.41)	1.07 (0.57, 2.01)	1.04 (0.82, 1.31)
Model 2	Ref	1.16 (0.62, 2.18)	1.39 (0.76, 2.55)	1.43 (0.77, 2.64)	1.18 (0.63, 2.23)	1.08 (0.85, 1.37)
Model 3	Ref	1.15 (0.61, 2.15)	1.36 (0.73, 2.51)	1.40 (0.75, 2.63)	1.17 (0.60, 2.26)	1.08 (0.84, 1.39)
*Non-cardia gastric cancer*						
No. of events	11	15	12	6	15	59
Model 1	Ref	1.35 (0.62, 2.96)	1.09 (0.48, 2.5)	0.53 (0.20, 1.44)	1.31 (0.60, 2.87)	1.00 (0.72, 1.37)
Model 2	Ref	1.32 (0.60, 2.90)	1.08 (0.47, 2.47)	0.53 (0.20, 1.45)	1.32 (0.60, 2.91)	1.00 (0.72, 1.38)
Model 3	Ref	1.39 (0.63, 3.07)	1.15 (0.49, 2.68)	0.56 (0.21, 1.55)	1.40 (0.62, 3.20)	1.02 (0.73, 1.42)
*Overlapping or unclassified gastric cancer*						
No. of events	21	15	18	12	15	81
Model 1	Ref	0.71 (0.36, 1.38)	0.86 (0.45, 1.62)	0.56 (0.28, 1.15)	0.70 (0.36, 1.37)	0.79 (0.60, 1.03)
Model 2	Ref	0.72 (0.37, 1.41)	0.89 (0.47, 1.69)	0.61 (0.30, 1.24)	0.75 (0.38, 1.48)	0.82 (0.62, 1.07)
Model 3	Ref	0.69 (0.35, 1.36)	0.85 (0.44, 1.63)	0.59 (0.29, 1.23)	0.72 (0.36, 1.47)	0.80 (0.60, 1.06)
**Nitrite intake from naturally-occurring animal sources**						
*Intake (mg/d)*	0.35 [0.30, 0.38]	0.46 [0.44, 0.49]	0.56 [0.53, 0.59]	0.68 [0.64, 0.72]	0.91 [0.82, 1.07]	0.56 [0.44, 0.72]
*Gastric cancer (combined)*						
No. of events	34	43	59	67	57	260
Model 1	Ref	1.18 (0.75, 1.86)	1.55 (1.01, 2.38)	1.67 (1.09, 2.56)	1.41 (0.91, 2.20)	1.21 (0.96, 1.52)
Model 2	Ref	1.14 (0.73, 1.80)	1.47 (0.95, 2.26)	1.55 (1.01, 2.38)	1.26 (0.81, 1.98)	1.13 (0.89, 1.42)
Model 3	Ref	1.14 (0.72, 1.80)	1.46 (0.94, 2.26)	1.54 (0.99, 2.40)	1.24 (0.77, 2.00)	1.11 (0.86, 1.43)
*Cardia gastric cancer*						
No. of events	12	21	27	33	27	120
Model 1	Ref	1.47 (0.72, 3.00)	1.66 (0.83, 3.32)	1.80 (0.91, 3.56)	1.41 (0.70, 2.84)	1.15 (0.82, 1.62)
Model 2	Ref	1.41 (0.69, 2.88)	1.54 (0.77, 3.09)	1.63 (0.82, 3.24)	1.20 (0.59, 2.45)	1.04 (0.73, 1.47)
Model 3	Ref	1.37 (0.67, 2.82)	1.48 (0.73, 2.98)	1.54 (0.77, 3.12)	1.10 (0.52, 2.34)	0.98 (0.67, 1.44)
*Non-cardia gastric cancer*						
No. of events	6	9	12	18	14	59
Model 1	Ref	1.51 (0.54, 4.26)	2.08 (0.77, 5.60)	3.16 (1.23, 8.15)	2.56 (0.95, 6.89)	1.50 (0.93, 2.41)
Model 2	Ref	1.46 (0.52, 4.11)	1.99 (0.74, 5.36)	2.98 (1.15, 7.71)	2.42 (0.89, 6.57)	1.44 (0.88, 2.36)
Model 3	Ref	1.60 (0.57, 4.55)	2.31 (0.84, 6.32)	3.62 (1.36, 9.63)	3.12 (1.09, 8.88)	1.63 (0.97, 2.74)
*Overlapping or unclassified gastric cancer*						
No. of events	16	13	20	16	16	81
Model 1	Ref	0.81 (0.39, 1.70)	1.27 (0.65, 2.48)	1.02 (0.50, 2.09)	1.05 (0.51, 2.18)	1.11 (0.74, 1.67)
Model 2	Ref	0.80 (0.38, 1.68)	1.24 (0.63, 2.44)	0.97 (0.47, 2.01)	0.97 (0.46, 2.03)	1.06 (0.70, 1.59)
Model 3	Ref	0.79 (0.37, 1.65)	1.19 (0.60, 2.37)	0.92 (0.44, 1.94)	0.88 (0.40, 1.95)	1.01 (0.65, 1.56)

Table 3 presents the rate of gastric cancer and subsites over 27 years of follow-up by intake of nitrate and nitrite from naturally-occurring animal sources

Hazard ratios (95% CIs) were estimated from Cox proportional hazards models with age as the underlying time scale. Nitrate and nitrite intakes were modelled categorically (quantiles) and as a logarithmic transformation base 2, where the hazard ratio represents the relative change in hazard associated with each doubling of the exposure (mg/d)

Model 1 included sex; Model 2 included sex, BMI, smoking status, smoking packyears, alcohol consumption, education level, physical activity level and living situation; Model 3 included all covariates in Model 2 and dietary confounders relevant for intake of nitrate and nitrite from naturally-occurring animal sources as exposure (intake of wholegrains, refined grains, vegetables, fruits, vegetable oils, sugar and confectionary, soft drinks, coffee, and tea)

Exposure intakes are median [IQR]. Quintiles were defined based on the distribution of the exposure within the study population, with cutoff points at the 20th, 40th, 60th, and 80th percentiles

For additive-permitted meat sources, a higher rate of overall GC for both nitrate and nitrite [HR_Q5vQ1_ nitrate 1.94 (1.19, 3.15); nitrite 2.08 (1.30, 3.35); Model 2, Table 4] was observed. Of the two GC subsites, higher point estimates were seen for non-cardia GC [HR_Q5vQ1_ nitrate 2.76 (1.00, 7.62); nitrite 3.34 (1.24, 9.01); Model 2] although a similar pattern was observed for cardia GC [HR_Q5vQ1_ nitrate 2.14 (0.99, 4.62); nitrite 2.23 (1.04, 4.80); Model 2].

**Table 4:** Hazards ratio of gastric cancer and subtypes by quintiles of nitrate and nitrite intake from additive-permitted meat sources

	Q1 *n* = 10,922	Q2 *n* = 10,922	Q3 *n* = 10,922	Q4 *n* = 10,922	Q5 *n* = 10,922	Log transformed *N* = 54,610
**Nitrate intake from additive-permitted meat sources**						
*Intake (mg/d)*	0.09 [0.06, 0.12]	0.18 [0.16, 0.21]	0.28 [0.26, 0.32]	0.43 [0.39, 0.48]	0.73 [0.62, 0.95]	0.28 [0.16, 0.48]
*Gastric cancer (combined)*						
No. of events	26	53	45	68	68	260
Model 1	Ref	1.99 (1.24, 3.18)	1.65 (1.02, 2.69)	2.37 (1.49, 3.78)	2.33 (1.44, 3.76)	1.24 (1.10, 1.39)
Model 2	Ref	1.90 (1.18, 3.04)	1.54 (0.94, 2.52)	2.11 (1.32, 3.37)	1.94 (1.19, 3.15)	1.17 (1.05, 1.31)
Model 3	Ref	1.89 (1.18, 3.04)	1.54 (0.94, 2.53)	2.11 (1.32, 3.40)	1.97 (1.20, 3.24)	1.18 (1.05, 1.33)
*Cardia gastric cancer*						
No. of events	9	23	21	29	38	120
Model 1	Ref	2.28 (1.05, 4.94)	1.91 (0.87, 4.20)	2.28 (1.06, 4.91)	2.7 (1.26, 5.77)	1.22 (1.03, 1.44)
Model 2	Ref	2.13 (0.98, 4.64)	1.74 (0.79, 3.84)	1.96 (0.91, 4.25)	2.14 (0.99, 4.62)	1.14 (0.96, 1.34)
Model 3	Ref	2.09 (0.96, 4.55)	1.68 (0.76, 3.72)	1.86 (0.86, 4.06)	1.99 (0.91, 4.35)	1.11 (0.94, 1.32)
*Non-cardia gastric cancer*						
No. of events	6	12	9	17	15	59
Model 1	Ref	2.08 (0.78, 5.56)	1.62 (0.57, 4.59)	3.18 (1.22, 8.25)	3.04 (1.12, 8.29)	1.35 (1.06, 1.72)
Model 2	Ref	1.96 (0.73, 5.26)	1.53 (0.54, 4.36)	2.95 (1.13, 7.71)	2.76 (1.00, 7.62)	1.32 (1.03, 1.69)
Model 3	Ref	2.12 (0.79, 5.69)	1.74 (0.61, 4.97)	3.53 (1.33, 9.33)	3.67 (1.30, 10.37)	1.43 (1.10, 1.85)
*Overlapping or unclassified gastric cancer*						
No. of events	11	18	15	22	15	81
Model 1	Ref	1.68 (0.79, 3.56)	1.43 (0.65, 3.13)	2.15 (1.02, 4.53)	1.54 (0.67, 3.53)	1.19 (0.98, 1.46)
Model 2	Ref	1.65 (0.77, 3.51)	1.37 (0.62, 3.01)	1.94 (0.91, 4.13)	1.29 (0.56, 3.00)	1.14 (0.93, 1.38)
Model 3	Ref	1.62 (0.76, 3.45)	1.33 (0.6, 2.95)	1.87 (0.87, 4.02)	1.25 (0.53, 2.96)	1.13 (0.92, 1.38)
**Nitrite intake from additive-permitted meat sources**						
*Intake (mg/d)*	0.02 [0.02, 0.03]	0.05 [0.04, 0.05]	0.07 [0.07, 0.08]	0.11 [0.10, 0.12]	0.19 [0.16, 0.24]	0.07 [0.04, 0.12]
*Gastric cancer (combined)*						
No. of events	27	48	52	59	74	260
Model 1	Ref	1.72 (1.07, 2.75)	1.83 (1.14, 2.92)	2.02 (1.27, 3.22)	2.48 (1.56, 3.95)	1.26 (1.13, 1.41)
Model 2	Ref	1.62 (1.01, 2.61)	1.68 (1.05, 2.70)	1.81 (1.13, 2.90)	2.08 (1.30, 3.35)	1.20 (1.07, 1.34)
Model 3	Ref	1.62 (1.01, 2.61)	1.69 (1.05, 2.71)	1.82 (1.13, 2.94)	2.11 (1.30, 3.43)	1.21 (1.07, 1.36)
*Cardia gastric cancer*						
No. of events	9	21	27	24	39	120
Model 1	Ref	2.06 (0.94, 4.50)	2.41 (1.13, 5.17)	1.95 (0.89, 4.26)	2.86 (1.35, 6.09)	1.21 (1.02, 1.42)
Model 2	Ref	1.90 (0.86, 4.16)	2.14 (0.99, 4.61)	1.66 (0.75, 3.65)	2.23 (1.04, 4.80)	1.12 (0.95, 1.32)
Model 3	Ref	1.86 (0.85, 4.08)	2.07 (0.96, 4.46)	1.58 (0.71, 3.49)	2.07 (0.95, 4.52)	1.10 (0.93, 1.30)
*Non-cardia gastric cancer*						
No. of events	6	12	12	11	18	59
Model 1	Ref	2.06 (0.77, 5.49)	2.15 (0.80, 5.76)	2.05 (0.74, 5.64)	3.61 (1.37, 9.56)	1.36 (1.07, 1.73)
Model 2	Ref	1.94 (0.72, 5.21)	2.02 (0.75, 5.46)	1.92 (0.69, 5.34)	3.34 (1.24, 9.01)	1.34 (1.05, 1.72)
Model 3	Ref	2.10 (0.78, 5.64)	2.29 (0.84, 6.23)	2.29 (0.82, 6.45)	4.36 (1.58, 11.99)	1.45 (1.12, 1.87)
*Overlapping or unclassified gastric cancer*						
No. of events	12	15	13	24	17	81
Model 1	Ref	1.28 (0.60, 2.74)	1.15 (0.52, 2.53)	2.18 (1.07, 4.46)	1.64 (0.75, 3.59)	1.28 (1.04, 1.56)
Model 2	Ref	1.26 (0.59, 2.71)	1.10 (0.50, 2.45)	2.06 (1.00, 4.26)	1.44 (0.65, 3.22)	1.23 (1.00, 1.50)
Model 3	Ref	1.24 (0.57, 2.67)	1.08 (0.48, 2.41)	2.01 (0.97, 4.19)	1.39 (0.61, 3.18)	1.22 (0.99, 1.50)

Table 4 presents the rate of gastric cancer and subsites over 27 years of follow-up by intake of nitrate and nitrite from additive-permitted meat sources

Hazard ratios (95% CIs) were estimated from Cox proportional hazards models with age as the underlying time scale. Nitrate and nitrite intakes were modelled categorically (quantiles) and as a logarithmic transformation base 2, where the hazard ratio represents the relative change in hazard associated with each doubling of the exposure (mg/d)

Model 1 included sex; Model 2 included sex, BMI, smoking status, smoking packyears, alcohol consumption, education level, physical activity level and living situation; Model 3 included all covariates in Model 2 and dietary confounders relevant for intake of nitrate and nitrite from additive-permitted meat sources as exposure (intake of wholegrains, refined grains, vegetables, fruits, vegetable oils, sugar and confectionary, soft drinks, coffee, and tea)

Exposure intakes are median [IQR]. Quintiles were defined based on the distribution of the exposure within the study population, with cutoff points at the 20th, 40th, 60th, and 80th percentiles

Nitrate intake from tap water (mg/d) at baseline was associated with a significantly higher rate of cardia GC [HR_Q5vQ1_ 1.95 (1.11, 3.43), Model 2, Table 5], but not non-cardia GC [HR_Q5vQ1_ 1.22 (0.55, 2.75)]. Similarly, in the sensitivity analysis modelling water nitrate concentration (mg/L) as a time-varying covariate over a 15-year window, participants exposed to the highest nitrate concentrations (median concentration: 5.1 mg/L), compared the lowest (median concentration: 0.8 mg/L) had a higher rate of cardia GC [HR_Q5vQ1_ 2.10 (1.04, 4.23); Model 2, Table 6), but not non-cardia GC [HR_Q5vQ1_ 0.60 (0.21, 1.74)].

**Table 5:** Hazards ratio of gastric cancer and subtypes by quintiles of tap water sourced nitrate intake

	Q1 *n* = 10,922	Q2 *n* = 10,922	Q3 *n* = 10,922	Q4 *n* = 10,922	Q5 *n* = 10,922	Log transformed *N* = 54,610
**Nitrate intake from tap water**						
*Intake (mg/d)*	0.04 [0.00, 0.10]	0.34 [0.25, 0.43]	0.79 [0.62, 0.94]	1.45 [1.14, 1.74]	3.26 [2.57, 4.84]	0.79 [0.25, 1.74]
*Gastric cancer (combined)*						
No. of events	60	56	41	46	57	260
Model 1	Ref	0.97 (0.67, 1.40)	0.73 (0.49, 1.09)	0.85 (0.58, 1.26)	1.14 (0.78, 1.67)	0.99 (0.96, 1.03)
Model 2	Ref	1.05 (0.73, 1.52)	0.80 (0.53, 1.19)	0.94 (0.63, 1.39)	1.23 (0.84, 1.80)	1.00 (0.97, 1.04)
Model 3	Ref	1.11 (0.76, 1.60)	0.88 (0.58, 1.34)	1.06 (0.70, 1.61)	1.46 (0.95, 2.23)	1.01 (0.98, 1.05)
*Cardia gastric cancer*						
No. of events	24	29	21	18	28	120
Model 1	Ref	1.35 (0.78, 2.32)	1.07 (0.59, 1.93)	0.99 (0.53, 1.84)	1.80 (1.02, 3.15)	1.02 (0.96, 1.07)
Model 2	Ref	1.49 (0.86, 2.56)	1.18 (0.65, 2.13)	1.13 (0.61, 2.10)	1.95 (1.11, 3.43)	1.03 (0.98, 1.09)
Model 3	Ref	1.51 (0.87, 2.61)	1.20 (0.65, 2.21)	1.15 (0.60, 2.20)	1.98 (1.12, 3.52)	1.03 (0.98, 1.09)
*Non-cardia gastric cancer*						
No. of events	12	15	8	10	14	59
Model 1	Ref	1.21 (0.57, 2.61)	0.64 (0.26, 1.57)	0.80 (0.34, 1.89)	1.16 (0.52, 2.60)	1.02 (0.94, 1.11)
Model 2	Ref	1.24 (0.58, 2.66)	0.65 (0.26, 1.62)	0.84 (0.35, 1.98)	1.22 (0.55, 2.75)	1.03 (0.94, 1.12)
Model 3	Ref	1.23 (0.56, 2.66)	0.63 (0.25, 1.69)	0.79 (0.32, 1.96)	1.10 (0.44, 2.75)	1.03 (0.94, 1.13)
*Overlapping or unclassified gastric cancer*						
No. of events	24	12	12	18	15	81
Model 1	Ref	0.48 (0.24, 0.96)	0.47 (0.23, 0.95)	0.70 (0.37, 1.32)	0.59 (0.30, 1.16)	0.94 (0.89, 0.99)
Model 2	Ref	0.53 (0.26, 1.07)	0.51 (0.25, 1.04)	0.75 (0.40, 1.42)	0.62 (0.32, 1.23)	0.96 (0.91, 1.01)
Model 3	Ref	0.55 (0.27, 1.10)	0.55 (0.27, 1.10)	0.80 (0.42, 1.53)	0.67 (0.33, 1.33)	0.96 (0.91, 1.02)

Table 5 presents the rate of gastric cancer and subsites over 27 years of follow-up by intake of tap water sourced nitrate

Hazard ratios (95% CIs) were estimated from Cox proportional hazards models with age as the underlying time scale. Nitrate intake was modelled categorically (quantiles) and as a logarithmic transformation base 2, where the hazard ratio represents the relative change in hazard associated with each doubling of the exposure (mg/d)

Model 1 included sex; Model 2 included sex, BMI, smoking status, smoking packyears, alcohol consumption, education level, physical activity level and living situation; Model 3 included all covariates in Model 2 and dietary confounders relevant for drinking water nitrate as exposure (intake of wholegrains, refined grains, red meat, processed meat, poultry, dairy, fish, vegetables, fruits, vegetable oils, sugar and confectionary and soft drinks)

Quintiles were defined based on the distribution of the exposure within the study population, with cutoff points at the 20th, 40th, 60th, and 80th percentiles

**Table 6:** Hazards ratio of gastric cancer and subtypes by quintiles of tap water nitrate concentration at baseline and from time-updated analyses

	Q1 *n* = 10,924	Q2 *n* = 10,923	Q3 *n* = 10,918	Q4 *n* = 10,933	Q5 *n* = 10,911	Log transformed *N* = 54,610
Nitrate concentration in tap water supplying the household, measured at baseline						
*Concentration (mg/L)*						
Median [IQR]	0.69 [0.51, 0.94]	1.35 [1.20, 1.51]	1.99 [1.87, 2.04]	2.41 [2.17, 2.85]	4.78 [3.76, 7.26]	1.99 [1.20, 2.85]
Range	0.02–1.08	1.08–1.73	1.73–2.13	2.13–3.06	3.06–33.79	0.02–33.79
*Gastric cancer (combined)*						
No. of events	48	53	36	67	56	260
Model 1	Ref	1.08 (0.73, 1.59)	0.75 (0.49, 1.16)	1.41 (0.97, 2.04)	1.17 (0.80, 1.72)	1.09 (0.98, 1.22)
Model 2	Ref	1.07 (0.72, 1.58)	0.74 (0.48, 1.15)	1.37 (0.95, 1.99)	1.12 (0.76, 1.66)	1.08 (0.96, 1.20)
Model 3	Ref	1.08 (0.73, 1.59)	0.75 (0.48, 1.16)	1.38 (0.95, 2.00)	1.12 (0.76, 1.65)	1.07 (0.96, 1.20)
*Cardia gastric cancer*						
No. of events	24	21	21	29	25	120
Model 1	Ref	0.85 (0.47, 1.53)	0.89 (0.49, 1.59)	1.23 (0.71, 2.11)	1.05 (0.60, 1.85)	1.09 (0.93, 1.28)
Model 2	Ref	0.85 (0.48, 1.54)	0.93 (0.51, 1.67)	1.24 (0.72, 2.14)	1.04 (0.59, 1.83)	1.08 (0.92, 1.27)
Model 3	Ref	0.86 (0.48, 1.55)	0.96 (0.53, 1.73)	1.28 (0.74, 2.21)	1.06 (0.61, 1.87)	1.08 (0.93, 1.27)
*Non-cardia gastric cancer*						
No. of events	12	13	9	16	9	59
Model 1	Ref	1.05 (0.48, 2.30)	0.74 (0.31, 1.76)	1.32 (0.62, 2.79)	0.74 (0.31, 1.77)	0.99 (0.79, 1.25)
Model 2	Ref	1.05 (0.48, 2.31)	0.75 (0.32, 1.80)	1.34 (0.63, 2.85)	0.74 (0.31, 1.77)	0.99 (0.79, 1.25)
Model 3	Ref	1.05 (0.48, 2.31)	0.73 (0.31, 1.76)	1.32 (0.62, 2.81)	0.72 (0.30, 1.73)	0.98 (0.78, 1.24)
*Overlapping or unclassified gastric cancer*						
No. of events	12	19	6	22	22	81
Model 1	Ref	1.56 (0.76, 3.21)	0.50 (0.19, 1.34)	1.84 (0.91, 3.73)	1.84 (0.91, 3.72)	1.17 (0.96, 1.42)
Model 2	Ref	1.52 (0.74, 3.13)	0.44 (0.16, 1.18)	1.68 (0.83, 3.40)	1.64 (0.81, 3.33)	1.14 (0.93, 1.39)
Model 3	Ref	1.07 (0.88, 1.30)	1.09 (0.89, 1.32)	1.14 (0.94, 1.38)	1.00 (0.82, 1.22)	1.01 (0.96, 1.07)

	Q1 *n* = 9127	Q2 *n* = 9096	Q3 *n* = 9116	Q4 *n* = 9122	Q5 *n* = 9095	Log transformed *N* = 45,556
Time-updated nitrate concentration in tap water supplying the household						
*Concentration (mg/L)*						
Median [IQR]	0.84 [0.71, 1.00]	1.38 [1.36, 1.44]	1.78 [1.76, 1.96]	2.38 [2.29, 2.88]	5.11 [4.20, 7.13]	1.78 [1.36, 2.88]
Range	0.30–1.18	1.19–1.68	1.69–2.20	2.20–3.28	3.28–48.30	0.30–48.30
*Gastric cancer (combined)*						
No. of events	31	38	34	37	37	177
Model 1	Ref	0.73 (0.44, 1.22)	0.86 (0.54, 1.38)	1.14 (0.74, 1.75)	1.36 (0.86, 2.13)	1.14 (0.98, 1.33)
Model 2	Ref	0.69 (0.42, 1.16)	0.84 (0.52, 1.35)	1.08 (0.70, 1.68)	1.26 (0.80, 1.99)	1.12 (0.96, 1.31)
Model 3	Ref	0.70 (0.42, 1.17)	0.86 (0.53, 1.38)	1.10 (0.71, 1.70)	1.27 (0.81, 2.01)	1.12 (0.96, 1.31)
*Cardia gastric cancer*						
No. of events	13	21	16	13	16	79
Model 1	Ref	1.35 (0.64, 2.85)	1.38 (0.67, 2.85)	1.04 (0.49, 2.22)	2.13 (1.06, 4.29)	1.20 (0.96, 1.51)
Model 2	Ref	1.40 (0.66, 2.97)	1.49 (0.72, 3.08)	1.08 (0.50, 2.30)	2.10 (1.04, 4.23)	1.19 (0.95, 1.48)
Model 3	Ref	1.43 (0.68, 3.03)	1.57 (0.76, 3.26)	1.12 (0.52, 2.40)	2.19 (1.08, 4.43)	1.20 (0.96, 1.49)
*Non-cardia gastric cancer*						
No. of events	10	6	6	10	8	40
Model 1	Ref	0.40 (0.13, 1.26)	0.36 (0.12, 1.15)	1.35 (0.63, 2.93)	0.61 (0.21, 1.77)	1.04 (0.75, 1.45)
Model 2	Ref	0.38 (0.12, 1.21)	0.35 (0.11, 1.12)	1.33 (0.61, 2.92)	0.60 (0.21, 1.74)	1.04 (0.74, 1.46)
Model 3	Ref	0.36 (0.11, 1.15)	0.34 (0.11, 1.07)	1.24 (0.56, 2.72)	0.56 (0.19, 1.63)	1.02 (0.72, 1.43)
*Overlapping or unclassified gastric cancer*						
No. of events	8	11	12	14	13	58
Model 1	Ref	0.42 (0.15, 1.17)	0.77 (0.35, 1.71)	1.03 (0.50, 2.15)	1.23 (0.58, 2.61)	1.13 (0.86, 1.47)
Model 2	Ref	0.35 (0.12, 0.97)	0.66 (0.30, 1.47)	0.87 (0.41, 1.82)	1.04 (0.48, 2.22)	1.09 (0.82, 1.44)
Model 3	Ref	0.35 (0.12, 0.98)	0.67 (0.30, 1.50)	0.88 (0.42, 1.84)	1.04 (0.48, 2.23)	1.09 (0.82, 1.44)

Table 6 presents the rate of gastric cancer and subsites over 27 years of follow-up by drinking water nitrate as a baseline concentration and a time-varying concentration (median [IQR]) estimated as the cumulative average nitrate concentration in the drinking water supplied to the participant’s residential addresses over 15 years prior to a diagnosis of gastric cancer or administrative censoring

Hazard ratios (95% CIs) were estimated from Cox proportional hazards models with age as the underlying time scale. Dietary nitrate and nitrite exposures were modelled categorically (quantiles) and as a logarithmic transformation base 2, where the hazard ratio represents the relative change in hazard associated with each doubling of the exposure (mg/L)

Model 1 included sex; Model 2 included sex, BMI, smoking status, smoking packyears, alcohol consumption, education level, physical activity level and living situation; Model 3 included all covariates in Model 2 and dietary confounders relevant for drinking water nitrate as exposure (intake of wholegrains, refined grains, red meat, processed meat, poultry, dairy, fish, vegetables, fruits, vegetable oils, sugar and confectionary and soft drinks, and for baseline concentration also intake of tap-water in g/d)

Quintiles were defined based on the distribution of the exposure within the study population, with cutoff points at the 20th, 40th, 60th, and 80th percentiles. Due to tied values at boundaries, quintile sizes may vary slightly from exactly 20% of the sample, resulting in unequal number of participants in each quintile

### Stratified analyses

**Fig. 1:**
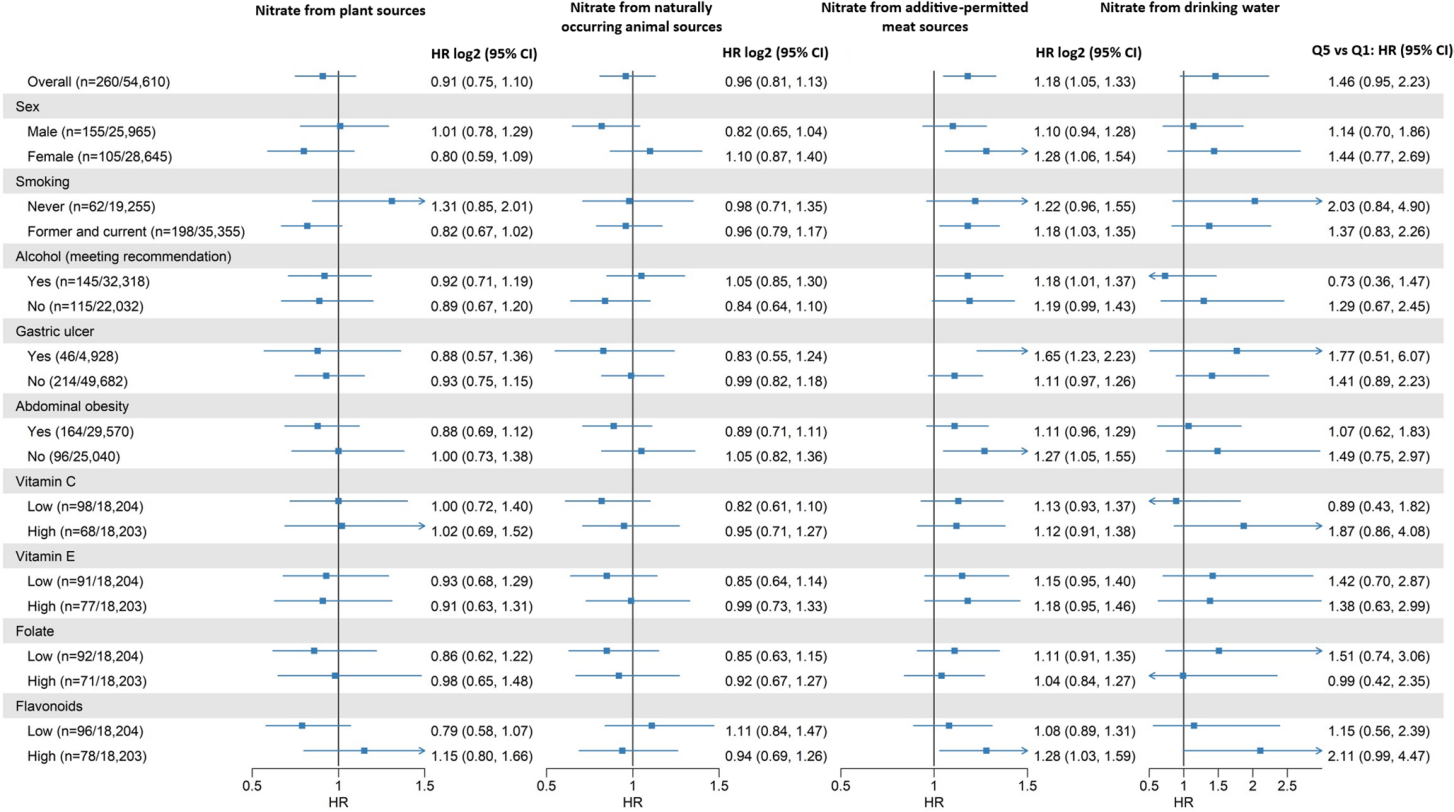
Forest plots depicting associations between intake of nitrate from plant sources, naturally-occurring animal sources, additive-permitted meat sources, and drinking water and rate of gastric cancer stratified by sex, smoking status (never vs. former and current), meeting the recommended maximum intake of alcohol (male: ≤ 14 units/week malesand female: ≤ 7 units/week emales; yes/no), a medical history of gastric ulcer (as a proxy for H. *pylori* infection; yes/no), abdominal obesity (yes/no), and dietary intakes of vitamin C, vitamin E, folate, and flavonoids. Dietary exposures were modeled on the log₂ scale to reflect hypothesized logarithmic associations (hazard ratios represent the relative change in hazard associated with each doubling of the exposure), while drinking water nitrate was modelled as quintile 5 versus quintile 1, hypothesizing higher risk only at the highest exposure levels. All analyses have age as the underlying time scale and uses Model 2 (adjusted for BMI, smoking status, smoking pack years, alcohol consumption, education level, physical activity level and living situation)

**Fig. 2:**
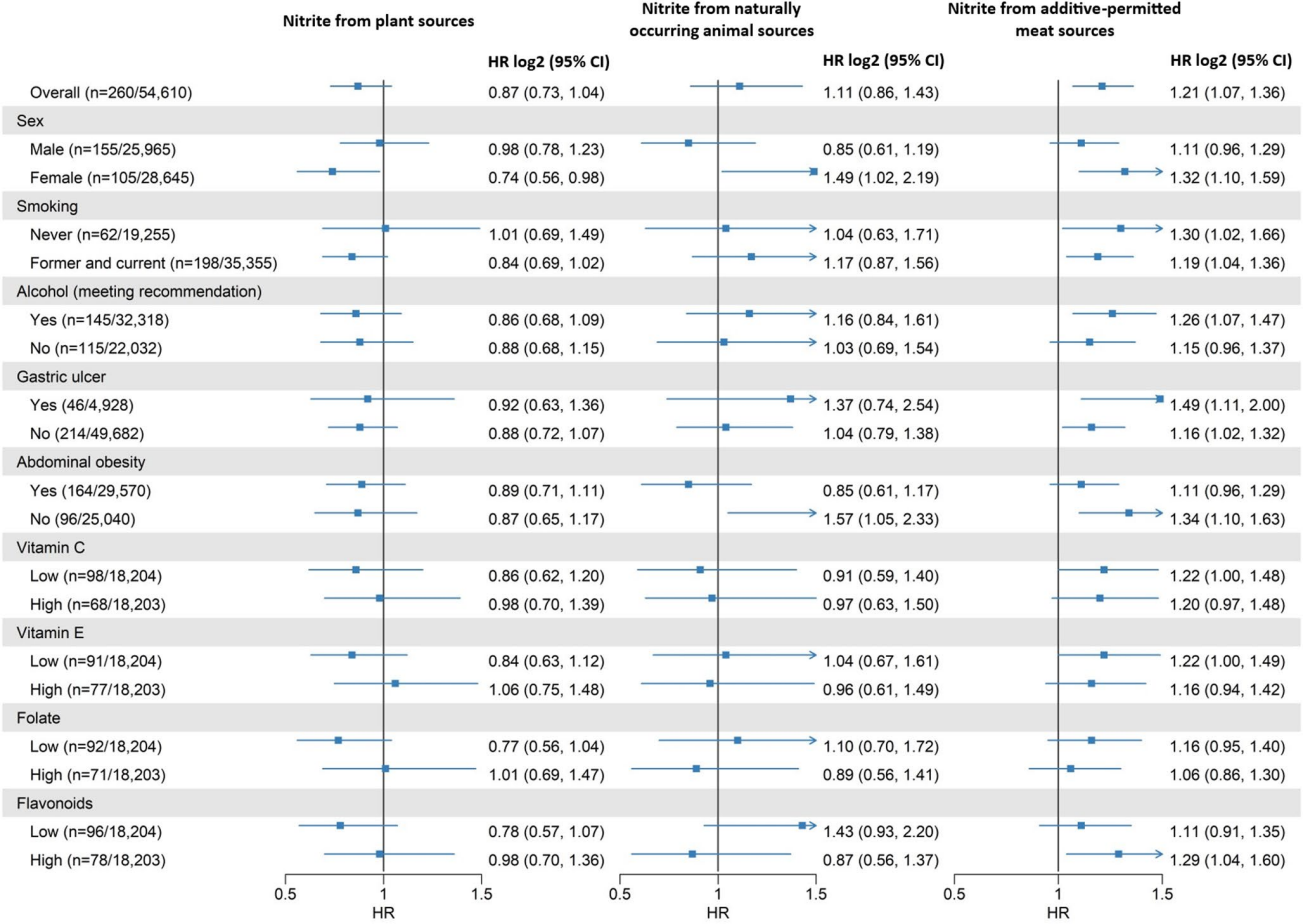
Forest plots depicting associations between intake of nitrite from plant sources, naturally-occurring animal sources and additive-permitted meat sources, and rate of gastric cancer stratified by sex, smoking status (never vs. former and current), meeting the recommended maximum intake of alcohol (male: ≤ 14 units/week and female: ≤ 7 units/week; yes/no), a medical history of gastric ulcer (as a proxy for *Helicobacter pylori* infection; yes/no), abdominal obesity (yes/no), and dietary intakes of vitamin C, vitamin E, folate, and flavonoids. Dietary exposures were modeled on the log₂ scale to reflect hypothesized logarithmic associations (hazard ratios represent the relative change in hazard associated with each doubling of the exposure). All analyses have age as the underlying time scale and uses Model 2 (adjusted for BMI, smoking status, smoking pack years, alcohol consumption, education level, physical activity level and living situation)

In the stratified analyses, associations for nitrate and nitrate intake and rate of GC were not significantly modified by factors influencing endogenous formation of *N*-nitrosamines or by established risk factors for GC (Figs. 1 and 2). However, some patterns were observed. For example, rates of GC appeared to differ for nitrate intake between participants reported to have never smoked and those who were former and current smokers – a pattern not observed for nitrite. Further, associations between additive-permitted meat sourced nitrate and nitrite and GC appeared to be more pronounced in females, in participants with a history of a gastric ulcer (as a proxy for *H. pylori* infection), and among participants without abdominal obesity. Similar patterns were observed for associations between nitrite, but not nitrate, from naturally-occurring animal sources and GC.

## Discussion

In this large prospective cohort of 54,610 participants with 260 cases of GC over 27 years of follow-up, we observed source-dependent and subsite-specific associations between dietary nitrate and nitrite intake and GC. Plant-derived nitrite intakes were associated with lower rates of non-cardia GC, while nitrite from naturally-occurring animal sources as well as both nitrate and nitrite from additive-permitted meat sources were associated with higher rates of non-cardia GC. Patterns appeared to be strongest among females, in participants with a history of gastric ulcer, and in participants without abdominal adiposity. Higher rates of cardia GC were observed among participants with the highest intake of nitrate from drinking water at baseline, and among participants exposed to the highest nitrate concentration levels in the time-updated analysis. These findings highlight the importance of distinguishing between dietary sources when evaluating nitrate and nitrite health effects, addressing a key limitation and calling into question the findings of previous reviews and meta-analyses examining total intakes [43–46].

While plant sources make up the majority of overall dietary nitrate and nitrite intake [30], few studies have specifically examined nitrate or nitrite from plant sources in relation to GC risk. Two studies examining associations between nitrate and nitrite intakes from vegetables and fruits reported no association [47–49], and one study reported a borderline significant trend toward a lower risk [50]. While our finding that higher intakes of plant-sourced nitrite were associated with lower rates of GC could be spurious, it is supported by studies on a food group level, observing higher vegetable intakes to be associated with lower risk of GC [51]. Mechanistically, nitric oxide (NO), generated from dietary nitrate and nitrite, exerts cytoprotective and anti-inflammatory effects, maintains gastric mucosal blood flow, enhances mucus secretion, and supports epithelial repair, all of which may protect against carcinogenesis [52, 53]. Bioactive components abundant in vegetables, including vitamins C and E and flavonoids, further inhibit *N*-nitrosamine and DNA adduct formation by mitigating oxidative stress [16, 54]. In addition, vegetable-derived phytochemicals may enhance detoxification enzyme activity, modulate immune function, and provide fiber-mediated protection [55, 56]. Given the generally low intake of plant-sourced nitrate and nitrite in our cohort [30], studies in populations with higher intake levels are needed to clarify the role of plant-sourced nitrate and nitrite intake in GC risk.

In this study, higher intakes of nitrite, but not nitrate, from naturally-occurring animal sources were associated with a higher rate of non-cardia GC. Nitrite can directly participate in *N*-nitrosamine formation under acidic gastric conditions, particularly in the presence of secondary amines derived from protein degradation [57], whereas dietary nitrate must first undergo bacterial reduction to nitrite in the oral cavity before becoming available for gastric nitrosation [10]. This distinction may be fundamental if it affects bioavailability, timing of exposure, or co-occurrence with nitrosation modifiers in specific foods. However, disentangling the independent effects of nitrite and nitrate from correlated dietary exposures, such as heme iron, amines, salt and antioxidants, remains a major methodological challenge [47]. Interpretation is further complicated by the heterogeneous cancer-risk profiles of the foods providing nitrite in this cohort [30]. Red meat and dairy products – the primary contributors of nitrite from naturally-occurring animal sources [30] – differ markedly in their nutritional properties and health effects. Whereas red meat is an established risk factor for non-cardia GC, providing a pro-carcinogenic environment through heme iron, heterocyclic amines, and polycyclic aromatic hydrocarbons [58], dairy intake has been inversely associated with several cancers [59–61], potentially due to protective components such as calcium, lactoferrin, and bioactive peptides [62]. While our finding is novel, we were unable to determine whether the observed nitrite association was driven primarily by red meat consumption or reflects genuine carcinogenic potential of nitrite even within otherwise-protective food matrices such as dairy – a critical distinction for public health interpretation. Mechanistic studies employing food-specific nitrate/nitrite exposures and the measurement of endogenous *N-*nitrosamine formation are essential to clarify these complex source-specific associations.

The consistent association observed in our study between both nitrate and nitrite from additive-permitted meat sources and rate of GC is noteworthy, especially in light of these compounds accounting for only 0.5% and 3.9% of total dietary nitrate and nitrite intake, respectively [30]. Notably, the low intake levels observed in our cohort, substantially below recent national estimates [63] and current regulatory limits [64, 65], likely reflect a combination of low consumption levels of additive-permitted meat products in the cohort and Denmark’s long-standing (since 1973) more stringent restrictions on the use of nitrate and nitrite as preservatives than by EU law and in the United States, resulting in lower concentrations of these compounds in Danish processed meat products [66]. Our observation of associations at intake levels well beneath established safety thresholds raises important questions about the adequacy of current regulatory limits, and adds to the limited but expanding pool of literature examining nitrate and nitrite intake from additive-permitted meat sources as a risk factor for GC [67–69]. While the strong correlation (Spearman’s ρ = 0.95) between intake of nitrate and nitrite from additive-permitted meat sources and the food group itself in this cohort [30] limits our ability to isolate the effects of nitrate and nitrite from those of other processed meat constituents, our findings are in line with the broader body of studies on processed meat intake and elevated GC risk [70, 71]. Numerous studies, including those from the EPIC cohorts [72], as well as evaluations by the World Cancer Research Fund [58], have established processed meat as a convincing risk factor for GC. Our results are consistent with this evidence and add weight to concerns that nitrate and nitrite preservatives, even at relatively low intake levels, may contribute to gastric carcinogenesis.

The WHO first established a guideline value of 50 mg/L as nitrate ion in drinking water in 1984 to protect against acute toxicity, particularly methemoglobinemia in bottle-fed infants [73]. In its most recent re-evaluation (2016), the WHO concluded that available evidence did not provide conclusive support for drinking water nitrate as a cancer risk factor, noting limitations in observational study designs and the omission of endogenous nitrosation as a potential contributing mechanism [74]. Since then, the body of epidemiological research examining the potential carcinogenic effects of nitrate in drinking water, including associations with GC, has expanded steadily. Recent studies have reported either null associations [75–78] or elevated risks [79, 80], with a meta-analysis suggesting that the magnitude of the association may depend on exposure level [79]. In this study, we observed a higher rate of cardia GC in the highest exposure quintile, both at baseline and in supplementary analyses using time-weighted nitrate concentration as the exposure metric. Importantly, these findings emerged at relatively low exposure levels and are, to our knowledge, novel, as previous studies on water nitrate have not conducted GC subsite analyses. By focusing solely on overall GC, earlier research may have missed subsite-specific associations – analyses that are challenging due to the need for accurate outcome classification and sufficient case numbers.

The differing associations between GC subsites for nitrate and nitrite sources were unexpected, given that carcinogenesis is hypothesized to occur via *N*-nitrosamine formation for all exposures. In this cohort, drinking water nitrate was associated with cardia GC, whereas nitrite from naturally occurring animal sources was primarily linked to non-cardia GC. While possibly a chance finding which should be interpreted with caution due to lower case numbers, this could reflect true etiological heterogeneity in nitrate- and nitrite-related gastric carcinogenesis, consistent with known variations in risk factors between these anatomical subsites [58]. Animal-sourced nitrate/nitrite are co-ingested with heme iron and amines that facilitate *N*-nitrosamine formation in the acidic distal stomach [81]. Conversely, water nitrate undergoes bacterial reduction to nitrite in the oral cavity [82], and drinking water nitrate has been associated with GC in prior studies [80, 83], though subsite-specific associations have, to our knowledge, not been examined. We hypothesize that salivary nitrite derived from water-sourced nitrate may facilitate nitrosation at the cardia, where bile acid reflux could further facilitate *N*-nitrosamine formation. However, this mechanism remains speculative and requires validation through mechanistic studies with relevant biomarkers, as well as large-scale epidemiological studies with source- and subsite-specific data.

Endogenous formation of *N*-nitrosamines is also shaped by external factors: smoking is associated with increased formation [84], whereas dietary components like vitamins, polyphenols, and flavonoids may inhibit the endogenous pathway [85, 86].These modifiers, however, likely act only on endogenous pathways, not on pre-formed *N*-nitrosamines. Our stratified analyses found no indication that associations between dietary nitrate and nitrite and GC were modified by smoking status or intake of potentially protective dietary factors. However, several limitations warrant a cautious interpretation: the timing of consumption of protective compounds may be critical [16] – an aspect we could not capture with our baseline dietary assessment – and statistical power for detecting interactions in stratified analyses was limited.

Regarding GC risk factors, in stratified analyses we observed that nitrate and nitrite intake from additive-permitted meat sources was associated with a higher rate of GC among females and participants without abdominal obesity, with similar non-significant patterns for naturally-occurring animal sources. Contrary to our hypothesis, stronger associations were not observed in higher-risk subgroups. Such null or attenuated effects in high-risk groups may reflect exposure saturation of baseline risk, effect modification, or competing mechanisms [87–91]. For example, although males consumed approximately double the intake of nitrate and nitrite from additive-permitted meat sources compared to females, they have a substantially higher baseline risk of GC due to hormonal, genetic, and behavioral factors which may outweigh any harmful effects of dietary *N*-nitrosamines [88, 92]. A similar pattern was seen for abdominal obesity, an established GC risk factor (95), where associations emerged only among those without obesity, maybe reflecting biological interaction or, more likely, the masking effect of high baseline risk.

*H. pylori* infection is a major risk factor for particularly non-cardia GC, promoting carcinogenesis through chronic inflammation and interactions with dietary and environmental exposures, including nitrosating agents [37, 93]. In our stratified analyses, using gastric ulcer diagnosis as a proxy for *H. pylori* infection, nitrate and nitrite intake from additive-permitted meat sources appeared to be more strongly associated with GC among participants with a history of gastric ulcers. These findings are consistent with previous epidemiologic evidence showing that the carcinogenic effects of processed meat may be amplified in the presence of *H. pylori*-related inflammation [72, 94], potentially through increased nitrosative stress [95]. However, the use of peptic ulcer diagnoses as a proxy for *H. pylori* infection has important limitations. Peptic ulcer subtypes differ in their underlying pathophysiology, and duodenal ulcer phenotypes in particular have been associated with a comparatively lower risk of GC [96]. Thus, this proxy may have selectively identified individuals with heterogeneous - or potentially lower - baseline GC risk, introducing misclassification and complicating interpretation of the observed interaction. In the absence of serological confirmation of *H. pylori* status, future studies incorporating validated infection biomarkers and ulcer subtyping are needed to clarify these interactions and guide targeted GC prevention.

This study’s prospective design, large sample size, long follow-up time, high follow-up rate (>99%) and noteworthy low rate of missing exposure and covariate data (<2%) are all important strengths of this study, supporting reliability of our findings. The water nitrate exposure assessment was based on high-quality data from a well-established national monitoring programme and characterized by minimal seasonal variation and absence of chemical disinfection, representing a major strength. However, nitrate concentrations in drinking water within this cohort were low (Q1: <1.06 mg/L, Q5: ≥3.06 mg/L) compared to the broader Danish population where approximately 10% are exposed to water nitrate levels exceeding 9 mg/L [97], with historically higher levels reported [98] – of particular relevance to this cohort of older participants. The absence of data on other potential carcinogens in water, such as pesticides, means residual confounding from co-exposures cannot be ruled out. Despite detailed confounder adjustments, of which many were validated and obtained from registries, residual confounding from unmeasured or imperfectly measured factors cannot be excluded, and we also acknowledge the possibility of chance findings due to multiple comparisons. The reliance on a single baseline dietary assessment to ascertain nitrate and nitrite exposures constitutes an important limitation [28], as a one-time measurement may not adequately capture long-term habitual intake, possibly attenuating risk estimates. Further, it was not possibility of account for variability in nitrate and nitrite content of foods due to cultivation, storage, and preparation, and the difficulty in isolating the effects of nitrate and nitrite from other components of additive-permitted meat sources due to strong correlations is also an important limitation of the study [30]. Outcome assessment was constrained by a large proportion of unspecified GC cases, often not reported on or excluded in epidemiological studies [72, 87], limiting our statistical power to explore subsite-specific associations. Linkage to the Danish Esophagogastric Cancer Group (DECG) database, containing detailed clinical, pathological, and histological information for GC cases nationwide, could have provided further valuable insights into potential differences in etiological risk profile [99]. Given the etiologic heterogeneity of GC, the absence of histology-based classifications (e.g., intestinal vs. diffuse type or Lauren classification) represents an important limitation, and future studies should, when possible, consider histological information. Stratified analyses, though prespecified and aligned with prior literature, were based on smaller subgroups and should be interpreted cautiously.

## Conclusion

Nitrate and nitrite intakes were differentially associated with rates of GC depending on their dietary source, with lower rates seen with high intakes of nitrite from plant sources and higher rates seen with higher intakes from drinking water as well as animal sources – both when naturally-occurring and as permitted additives. More research is needed to further elucidate these source- and subsite-specific findings. Higher rates observed at nitrate levels below regulatory thresholds for drinking water and for relatively low additive-permitted meat intakes calls for re-evaluation of current legislation while higher risks seen for naturally-occurring additive sourced nitrite are novel and warrant further investigation.

## Supplementary Information

Below is the link to the electronic supplementary material.

Supplementary material 1 (DOCX 203.2 kb)

## Data Availability

The datasets presented in this article are not readily available due to the sensitive nature of the data collected for this study. Requests to access the dataset from qualified researchers trained in human subject confidentiality protocols may be sent to the Diet Cancer and Health Steering Committee at the Danish Cancer Institute. Requests to access the datasets should be directed to dchdata@cancer.dk.
