# Supplementary material for: Source-specific nitrate and nitrite intake and associations with gastric cancer in the Danish Diet, Cancer and Health Cohort
Source: Eur J Epidemiol. 2026 Apr 30;41(5):569–86. doi: 10.1007/s10654-026-01390-6 (PMC13332898; doi:10.1007/s10654-026-01390-6)
Supplement: Supplementary file 1 — Supplementary material 1 (DOCX 203.2 kb) [file 10654_2026_1390_MOESM1_ESM.docx]

SUPPLEMENTARY MATERIAL

**Source-specific nitrate and nitrite intake and association with gastric cancer in**

**the Danish Diet, Cancer and Health Cohort**

Dorit W. Erichsen et al.

Version date 25 February 2026


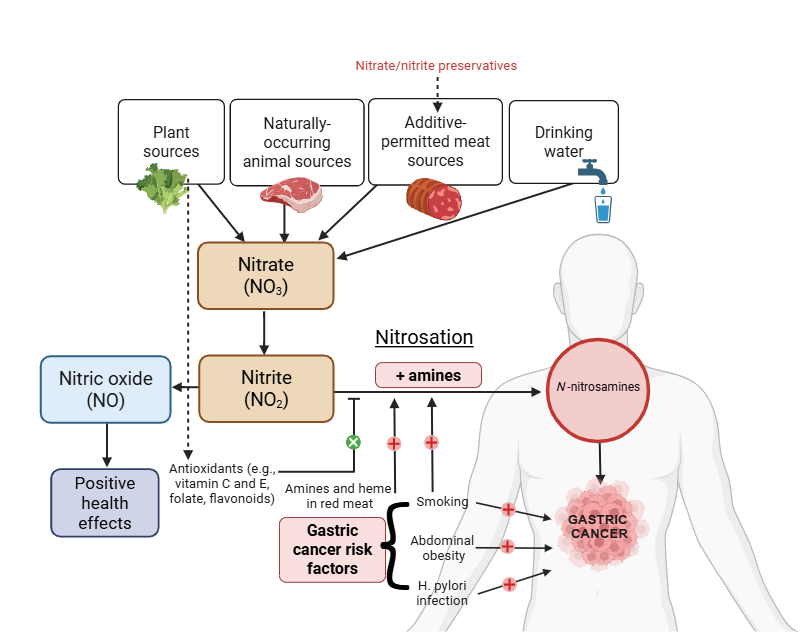


**Supplementary Figure 1**. Conceptual model of nitrate and nitrite sources, mechanistic pathways and dual health effects


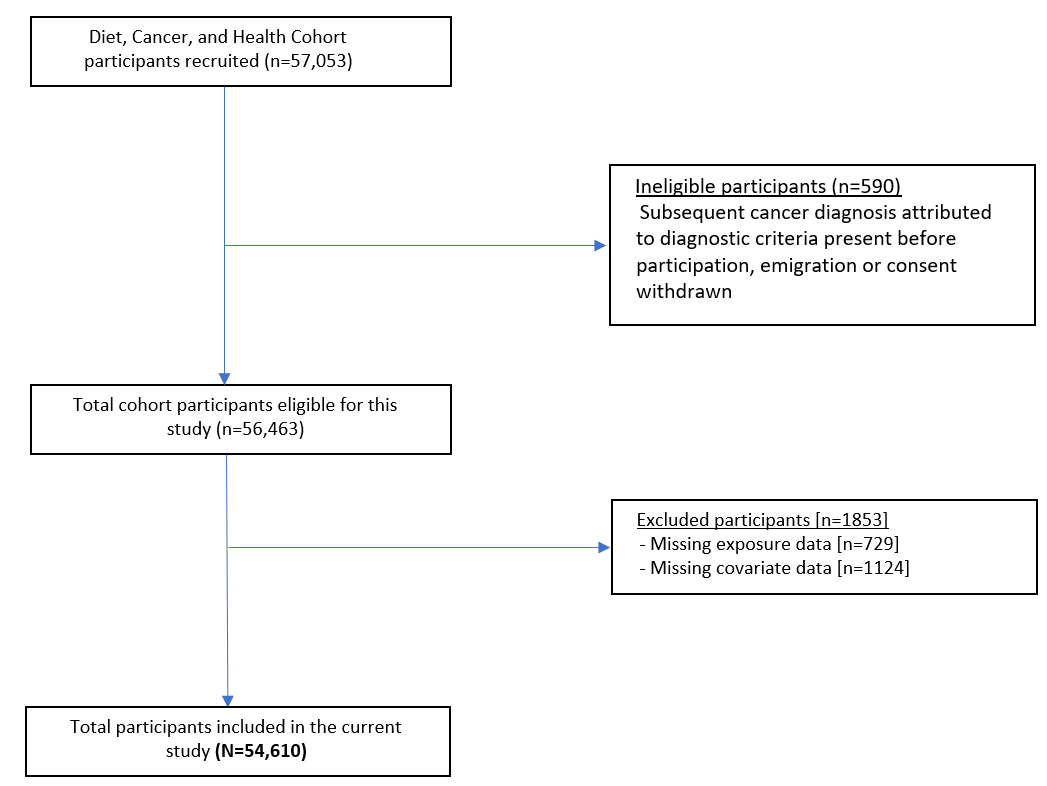


**Supplementary Figure 2.** CONSORT flow diagram of the Diet, Cancer and Health Cohort participants included in the present study

**Supplementary Table 1**. Dietary characteristics of the study population by quintile of source-specific nitrate intakes

|  | **Total participants** | **Plant-sourced nitrate** | | **Naturally occurring animal-sourced nitrate** | | **Additive-permitted meat-sourced nitrate** | | **Tap water-sourced nitrate** | |
| --- | --- | --- | --- | --- | --- | --- | --- | --- | --- |
|  | **N=54,610** | **Q1 (n=10,922)** | **Q5 (n=10,922)** | **Q1 (n=10,922)** | **Q5 (n=10,922)** | **Q1 (n=10,922)** | **Q5 (n=10,922)** | **Q1 (n=10,922)** | **Q5 (n=10,918)** |
| **Dietary intake** |  |  |  |  |  |  |  |  |  |
| Energy (kcal/d) | 2270.6 [1877.9, 2718.6] | 1946.2 [1598.2, 2338.0] | 2573.6 [2173.1, 3059.5] | 1816.9 [1537.1, 2148.6] | 2661.6 [2258.3, 3144.3] | 1916.3 [1588.6, 2283.7] | 2791.8 [2375.8, 3258.9] | 2349.2 [1952.3, 2794.8] | 2183.0 [1804.3, 2627.5] |
| Vegetables (g/d) | 309.9 [232.2, 403.1] | 183.2 [142.3, 222.3] | 474.7 [403.8, 564.7] | 257.0 [185.7, 344.2] | 358.4 [276.4, 457.4] | 305.0 [220.6, 407.3] | 333.6 [250.7, 428.9] | 289.3 [215.9, 377.1] | 323.8 [239.8, 425.3] |
| Fruits (g/d) | 169.8 [92.9, 280.0] | 86.7 [39.2, 152.7] | 283.0 [177.4, 432.2] | 140.3 [67.0, 244.9] | 219.4 [132.1, 346.5] | 196.1 [112.8, 322.6] | 148.1 [75.7, 250.9] | 131.6 [63.1, 227.7] | 196.2 [113.7, 320.4] |
| Wholegrains (g/d) | 121.6 [81.2, 168.9] | 103.5 [65.5, 147.0] | 152.9 [104.1, 203.9] | 112.5 [68.3, 163.0] | 135.2 [98.6, 186.3] | 115.2 [72.2, 164.3] | 152.7 [106.0, 202.9] | 116.5 [70.4, 165.2] | 124.6 [83.1, 170.2] |
| Refined grains (g/d) | 45.8 [29.3, 72.3] | 43.3 [26.1, 80.9] | 48.2 [31.4, 71.1] | 38.1 [23.1, 58.5] | 49.6 [32.4, 76.2] | 35.3 [22.3, 53.0] | 59.6 [38.8, 97.8] | 49.4 [31.0, 86.2] | 42.3 [26.9, 63.2] |
| Red meat (g/d) | 81.4 [58.6, 111.4] | 74.8 [54.6, 100.4] | 81.7 [55.9, 116.3] | 56.5 [42.5, 70.4] | 101.4 [73.4, 138.7] | 55.1 [38.5, 74.7] | 113.0 [85.0, 148.6] | 92.8 [67.8, 123.2] | 72.3 [51.7, 98.8] |
| Processed meat (g/d) | 21.6 [12.2, 35.5] | 22.4 [12.8, 36.6] | 19.1 [9.6, 33.0] | 15.9 [8.5, 26.4] | 24.6 [14.2, 39.4] | 6.6 [4.5, 8.7] | 52.1 [42.5, 67.4] | 26.0 [15.0, 41.3] | 18.5 [9.9, 30.6] |
| Poultry (g/d) | 17.9 [10.3, 27.6] | 12.5 [6.8, 19.9] | 22.8 [13.4, 35.3] | 12.9 [6.8, 21.1] | 21.0 [12.8, 32.9] | 15.4 [8.1, 25.8] | 19.6 [11.3, 30.6] | 17.1 [9.4, 26.2] | 18.3 [10.3, 29.0] |
| Fish (g/d) | 38.2 [25.4, 55.3] | 28.8 [18.8, 42.1] | 47.5 [31.8, 68.2] | 28.3 [18.6, 41.1] | 47.7 [32.2, 67.9] | 33.1 [21.4, 49.3] | 43.1 [28.1, 62.1] | 35.6 [23.2, 51.6] | 39.3 [26.1, 57.6] |
| Dairy (g/d) | 306.2 [165.7, 570.7] | 263.0 [119.6, 547.1] | 354.0 [215.1, 615.8] | 137.5 [74.6, 285.2] | 537.0 [379.6, 795.6] | 286.2 [148.3, 514.8] | 329.5 [178.7, 608.1] | 288.9 [144.7, 564.4] | 302.6 [163.1, 557.9] |
| Butter (g/d) | 9.2 [1.0, 20.0] | 10.5 [2.2, 19.8] | 7.5 [0.4, 20.3] | 6.1 [0.3, 16.2] | 10.3 [1.2, 21.4] | 6.0 [0.3, 16.1] | 15.1 [2.6, 25.6] | 12.2 [2.0, 22.1] | 6.8 [0.6, 17.8] |
| Vegetable oils (g/d) | 4.7 [1.2, 8.9] | 1.3 [0.5, 2.8] | 9.0 [4.7, 13.4] | 1.9 [0.6, 5.9] | 5.3 [1.6, 11.3] | 5.1 [1.0, 9.1] | 2.7 [1.1, 8.4] | 2.6 [0.9, 7.5] | 4.8 [1.0, 9.1] |
| Confectionery (g/d) | 49.0 [28.5, 81.1] | 41.8 [22.9, 72.6] | 53.3 [31.5, 87.6] | 40.7 [22.2, 70.2] | 56.3 [33.9, 92.0] | 41.6 [23.2, 71.3] | 57.5 [33.3, 98.2] | 49.4 [27.8, 84.7] | 45.5 [26.1, 76.0] |
| Soft drink (g/d) | 16.4 [3.3, 45.0] | 16.4 [3.3, 85.7] | 6.6 [0.0, 28.6] | 6.6 [0.0, 28.6] | 16.4 [3.3, 57.2] | 6.6 [0.0, 16.4] | 28.6 [6.6, 85.7] | 16.4 [3.3, 85.7] | 9.9 [0.0, 28.6] |
| Alcohol (g/d) | 12.9 [5.9, 31.1] | 12.1 [3.6, 32.3] | 12.7 [6.0, 29.3] | 11.0 [3.3, 26.9] | 13.3 [6.3, 30.5] | 10.3 [3.2, 21.0] | 16.5 [7.4, 36.8] | 16.0 [7.0, 36.6] | 11.2 [3.9, 23.0] |
| Water (l/d) | 1.7 [1.3, 2.1] | 1.6 [1.1, 2.0] | 1.8 [1.4, 2.3] | 1.7 [1.3, 2.2] | 1.8 [1.3, 2.2] | 1.8 [1.3, 2.2] | 1.6 [1.2, 2.1] | 1.3 [0.9, 1.6] | 2.2 [1.8, 2.6] |
| Tea (g/d) | 85.7 [3.3, 500.0] | 16.4 [0.0, 200.0] | 200.0 [16.4, 500.0] | 85.7 [3.3, 500.0] | 157.1 [16.4, 500.0] | 200.0 [16.4, 500.0] | 16.4 [3.3, 200.0] | 16.4 [3.3, 500.0] | 85.7 [6.6, 500.0] |
| Coffee (g/d) | 900.0 [500.0, 1300.0] | 900.0 [500.0, 1300.0] | 500.0 [500.0, 900.0] | 900.0 [500.0, 1300.0] | 900.0 [500.0, 1300.0] | 500.0 [500.0, 900.0] | 900.0 [500.0, 1300.0] | 900.0 [500.0, 1300.0] | 900.0 [500.0, 900.0] |
| Polyphenols (mg/d) | 1606.0 [1264.4, 1947.9] | 1425.9 [1088.4, 1764.7] | 1767.9 [1431.1, 2152.9] | 1521.4 [1165.3, 1863.1] | 1679.3 [1343.0, 2051.6] | 1543.5 [1194.4, 1913.2] | 1680.9 [1332.4, 2005.1] | 1631.0 [1270.5, 1951.8] | 1591.5 [1233.4, 1964.4] |
| Folic acid (µg/d) | 367.1 [289.1, 462.0] | 272.4 [218.1, 348.6] | 473.1 [398.1, 567.7] | 299.7 [234.1, 385.9] | 444.5 [362.3, 542.0] | 354.4 [272.9, 453.7] | 394.9 [315.4, 489.5] | 342.9 [269.5, 431.7] | 382.6 [298.3, 485.2] |
| Vitamin C (mg/d) | 125.1 [86.4, 183.8] | 74.2 [52.2, 115.5] | 184.6 [140.6, 259.6] | 108.0 [70.3, 165.0] | 151.5 [108.7, 215.5] | 137.4 [92.3, 214.4] | 118.5 [83.7, 168.3] | 104.9 [72.6, 152.5] | 140.8 [96.3, 215.7] |
| Vitamin E (mg α-TE/d) | 11.9 [8.3, 17.8] | 8.6 [6.0, 14.4] | 15.3 [11.2, 21.5] | 9.7 [6.5, 16.1] | 14.3 [10.2, 20.2] | 11.4 [7.4, 18.0] | 13.1 [9.3, 18.6] | 10.7 [7.6, 16.0] | 13.0 [8.6, 19.2] |
